# 3-[1-(4-Chloro­phen­yl)eth­yl]-1,3-thia­zinane-2-thione

**DOI:** 10.1107/S1600536811002078

**Published:** 2011-01-29

**Authors:** Yuan-Yuan Gong, Peng Zhang, Ming-Hui Wang

**Affiliations:** a College of Chemistry and Molecular Engineering, Qingdao University of Science and Technology, Qingdao 266042, People’s Republic of China

## Abstract

In the title compound, C_12_H_14_ClNS_2_, the thia­zole ring adopts an envelope conformation; the basal plane is nearly perpendicular to the benzene ring at a dihedral angle of 85.72 (5)°. Weak inter­molecular C—H⋯S hydrogen bonding is present in the crystal structure.

## Related literature

For the biological activity of thia­zole compounds, see: Amir *et al.* (2006 ▶). For a related structure, see: Cunico *et al.* (2007 ▶). 
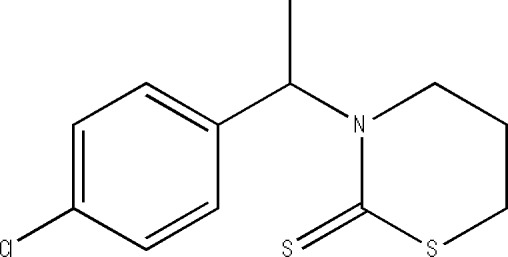


## Experimental

### 

#### Crystal data

C_12_H_14_ClNS_2_
*M* *_r_* = 271.81
Orthorhombic, 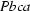

*a* = 11.260 (2) Å
*b* = 11.888 (2) Å
*c* = 18.978 (4) Å
*V* = 2540.5 (9) Å^3^
*Z* = 8
Mo *K*α radiation
μ = 0.60 mm^−1^
*T* = 113 K
0.18 × 0.14 × 0.12 mm

#### Data collection

Rigaku Saturn diffractometer
Absorption correction: multi-scan (*CrystalClear*; Rigaku, 2005 ▶) *T*_min_ = 0.900, *T*_max_ = 0.931
16988 measured reflections
2932 independent reflections
2605 reflections with *I* > 2σ(*I*)
*R*_int_ = 0.050

#### Refinement

*R*[*F* ^2^ > 2σ(*F* ^2^)] = 0.038
*wR*(*F* ^2^) = 0.089
*S* = 1.11
2925 reflections
147 parameters
H-atom parameters constrained
Δρ_max_ = 0.29 e Å^−3^
Δρ_min_ = −0.26 e Å^−3^

### 

Data collection: *CrystalClear* (Rigaku, 2005 ▶); cell refinement: *CrystalClear*; data reduction: *CrystalClear*; program(s) used to solve structure: *SHELXTL* (Sheldrick, 2008 ▶); program(s) used to refine structure: *SHELXTL*; molecular graphics: *SHELXTL*; software used to prepare material for publication: *SHELXTL*.

## Supplementary Material

Crystal structure: contains datablocks I, global. DOI: 10.1107/S1600536811002078/xu5140sup1.cif

Structure factors: contains datablocks I. DOI: 10.1107/S1600536811002078/xu5140Isup2.hkl

Additional supplementary materials:  crystallographic information; 3D view; checkCIF report

## Figures and Tables

**Table 1:** Hydrogen-bond geometry (Å, °)

*D*—H⋯*A*	*D*—H	H⋯*A*	*D*⋯*A*	*D*—H⋯*A*
C9—H9*A*⋯S2^i^	0.97	2.85	3.773 (2)	158
C10—H10*B*⋯S2^ii^	0.97	2.77	3.701 (2)	160

Symmetry codes: (i) 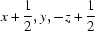; (ii) 
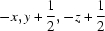
.

**Fig. 1.**
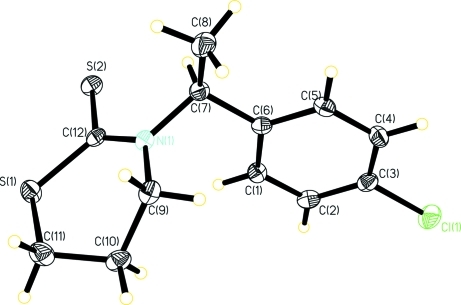
The molecular structure of (I), with displacement ellipsoids drawn at the 40% probability level.

## supplementary crystallographic information

### Comment

Recently, compounds containing a 1,3-thiazinane group have attracted much interest because the 1,3-thiazinane ring system are well known as its efficient insecticidal activity for a wide variety of crops (Amir *et al.*, 2006). The title compound (I) was synthesized as a new compound with better biological activity. We report here the crystal structure of (I).

In (I) all bond lengths and angles are normal and in a good agreement with those reported previously (Cunico *et al.*, 2007). The thiazole ring is in and envelope conformation with the –CH_2_– group bonded to the S1 atom forming the flap. The 1,3-thiazinane-2-thione ring forms two dihedral angles are 85.99 (2)° [S1/S2/N1/C7/C9/C11/C12] and 77.68 (2)° [N1/C9/C10/C11/C12] with the benzene ring respectively. The crystal structure is stabilized by weak intermolecular C–H···S hydrogen bonds.

### Experimental

1,3-Thiazinane-2-thione 1.33 g (10.0 mmol) and deacid reagent potassium carbonate 1.38 g (5.0 mmol) were added in a flask equipped with stirrer, the solvent acetonitrile (20 ml) was added and the mixture was stirred for 0.5 h. Then 1-chloro-4-(1-chloroethyl)benzene 1.74 g (10.0 mmol) was added dropwising within 2 h at 333 K. The mixture was stirred for 8 h at 433 K. Upon cooling at room temperature. then the solid was filterred, the filter-cake was washed twice by acetonitrile. Crystallized from methanol to afford the title compound 2.0 g (74% yield) Single crystals suitable for X-ray measurement were obtained by recrystallization from the mixture of acetone and methanol at room temperature.

### Refinement

H atoms were placed in calculated positions, with C—H = 0.93–0.98 Å, and included in the final cycles of refinement using a riding model with *U*_iso_(H) = 1.5*U*_eq_(C) for methyl H atoms and 1.2*U*_eq_(C) for the others.

### Crystal data

**Table:**

C_12_H_14_ClNS_2_	*F*(000) = 1136
*M**_r_* = 271.81	*D*_x_ = 1.421 Mg m^−^^3^
Orthorhombic, *P**b**c**a*	Mo *K*α radiation, λ = 0.71073 Å
Hall symbol: -P 2ac 2ab	Cell parameters from 5973 reflections
*a* = 11.260 (2) Å	θ = 2.2–27.5°
*b* = 11.888 (2) Å	µ = 0.60 mm^−^^1^
*c* = 18.978 (4) Å	*T* = 113 K
*V* = 2540.5 (9) Å^3^	Block, colorless
*Z* = 8	0.18 × 0.14 × 0.12 mm

### Data collection

**Table:**

Rigaku Saturn diffractometer	2932 independent reflections
Radiation source: rotating anode	2605 reflections with *I* > 2σ(*I*)
confocal	*R*_int_ = 0.050
ω scans	θ_max_ = 27.5°, θ_min_ = 2.2°
Absorption correction: multi-scan (*CrystalClear*; Rigaku, 2005)	*h* = −14→14
*T*_min_ = 0.900, *T*_max_ = 0.931	*k* = −7→15
16988 measured reflections	*l* = −24→24

### Refinement

**Table:**

Refinement on *F*^2^	Secondary atom site location: difference Fourier map
Least-squares matrix: full	Hydrogen site location: inferred from neighbouring sites
*R*[*F*^2^ > 2σ(*F*^2^)] = 0.038	H-atom parameters constrained
*wR*(*F*^2^) = 0.089	*w* = 1/[σ^2^(*F*_o_^2^) + (0.0383*P*)^2^ + 0.9612*P*] where *P* = (*F*_o_^2^ + 2*F*_c_^2^)/3
*S* = 1.11	(Δ/σ)_max_ = 0.002
2925 reflections	Δρ_max_ = 0.29 e Å^−^^3^
147 parameters	Δρ_min_ = −0.26 e Å^−^^3^
0 restraints	Extinction correction: *SHELXTL* (Sheldrick, 2008), Fc^*^=kFc[1+0.001xFc^2^λ^3^/sin(2θ)]^-1/4^
Primary atom site location: structure-invariant direct methods	Extinction coefficient: 0.0031 (5)

### Special details

**Table:**

Geometry. All e.s.d.'s (except the e.s.d. in the dihedral angle between two l.s. planes) are estimated using the full covariance matrix. The cell e.s.d.'s are taken into account individually in the estimation of e.s.d.'s in distances, angles and torsion angles; correlations between e.s.d.'s in cell parameters are only used when they are defined by crystal symmetry. An approximate (isotropic) treatment of cell e.s.d.'s is used for estimating e.s.d.'s involving l.s. planes.
Refinement. Refinement of *F*^2^ against ALL reflections. The weighted *R*-factor *wR* and goodness of fit *S* are based on *F*^2^, conventional *R*-factors *R* are based on *F*, with *F* set to zero for negative *F*^2^. The threshold expression of *F*^2^ > σ(*F*^2^) is used only for calculating *R*-factors(gt) *etc*. and is not relevant to the choice of reflections for refinement. *R*-factors based on *F*^2^ are statistically about twice as large as those based on *F*, and *R*- factors based on ALL data will be even larger.

### Fractional atomic coordinates and isotropic or equivalent isotropic displacement parameters (Å^2^)

**Table:**

	*x*	*y*	*z*	*U*_iso_*/*U*_eq_	
Cl1	−0.02163 (5)	0.84121 (4)	0.54940 (3)	0.03747 (15)	
S1	0.12139 (4)	0.46585 (4)	0.16831 (2)	0.02721 (14)	
S2	−0.00573 (4)	0.34132 (4)	0.27142 (2)	0.02514 (13)	
N1	0.17516 (12)	0.47749 (11)	0.30690 (7)	0.0190 (3)	
C1	0.03561 (15)	0.61986 (15)	0.39541 (9)	0.0223 (4)	
H1	0.0095	0.6085	0.3495	0.027*	
C2	−0.00917 (15)	0.70925 (15)	0.43370 (10)	0.0244 (4)	
H2	−0.0652	0.7573	0.4140	0.029*	
C3	0.03060 (16)	0.72615 (14)	0.50171 (10)	0.0247 (4)	
C4	0.11393 (16)	0.65588 (15)	0.53172 (9)	0.0263 (4)	
H4	0.1405	0.6684	0.5774	0.032*	
C5	0.15754 (16)	0.56606 (15)	0.49268 (9)	0.0239 (4)	
H5	0.2135	0.5183	0.5127	0.029*	
C6	0.11905 (14)	0.54630 (13)	0.42412 (9)	0.0191 (3)	
C7	0.15969 (15)	0.44518 (14)	0.38191 (9)	0.0211 (4)	
H7	0.0951	0.3900	0.3836	0.025*	
C8	0.27095 (17)	0.38623 (17)	0.40901 (10)	0.0317 (4)	
H8A	0.2939	0.3284	0.3765	0.047*	
H8B	0.2548	0.3533	0.4542	0.047*	
H8C	0.3342	0.4399	0.4135	0.047*	
C9	0.27378 (15)	0.55691 (15)	0.29311 (9)	0.0229 (4)	
H9A	0.3458	0.5144	0.2843	0.027*	
H9B	0.2868	0.6025	0.3348	0.027*	
C10	0.25045 (16)	0.63354 (15)	0.23103 (9)	0.0258 (4)	
H10A	0.3144	0.6878	0.2270	0.031*	
H10B	0.1771	0.6745	0.2387	0.031*	
C11	0.24125 (17)	0.56687 (17)	0.16328 (10)	0.0305 (4)	
H11A	0.3155	0.5278	0.1547	0.037*	
H11B	0.2273	0.6178	0.1242	0.037*	
C12	0.10630 (14)	0.43289 (14)	0.25731 (9)	0.0194 (3)	

### Atomic displacement parameters (Å^2^)

**Table:**

	*U*^11^	*U*^22^	*U*^33^	*U*^12^	*U*^13^	*U*^23^
Cl1	0.0465 (3)	0.0281 (3)	0.0379 (3)	0.0064 (2)	0.0101 (2)	−0.0076 (2)
S1	0.0275 (3)	0.0321 (3)	0.0220 (2)	−0.00856 (19)	−0.00050 (18)	−0.00137 (18)
S2	0.0221 (2)	0.0233 (2)	0.0301 (2)	−0.00669 (17)	−0.00089 (18)	−0.00064 (17)
N1	0.0159 (7)	0.0190 (7)	0.0222 (7)	−0.0002 (5)	−0.0002 (6)	−0.0020 (6)
C1	0.0184 (8)	0.0250 (8)	0.0236 (9)	−0.0008 (7)	−0.0032 (7)	−0.0004 (7)
C2	0.0185 (8)	0.0219 (8)	0.0327 (10)	0.0015 (7)	0.0007 (7)	0.0034 (7)
C3	0.0264 (9)	0.0197 (8)	0.0282 (9)	−0.0021 (7)	0.0084 (7)	−0.0006 (7)
C4	0.0304 (10)	0.0293 (9)	0.0192 (8)	−0.0021 (8)	0.0022 (7)	0.0013 (7)
C5	0.0253 (9)	0.0246 (8)	0.0220 (8)	0.0020 (7)	−0.0003 (7)	0.0042 (7)
C6	0.0161 (8)	0.0198 (8)	0.0215 (8)	−0.0023 (6)	0.0012 (7)	0.0028 (7)
C7	0.0213 (9)	0.0204 (8)	0.0215 (8)	−0.0001 (7)	−0.0020 (7)	0.0007 (7)
C8	0.0342 (11)	0.0283 (9)	0.0325 (10)	0.0106 (8)	−0.0070 (8)	−0.0031 (8)
C9	0.0159 (8)	0.0247 (8)	0.0280 (9)	−0.0043 (7)	0.0015 (7)	−0.0042 (7)
C10	0.0214 (9)	0.0206 (8)	0.0353 (10)	−0.0045 (7)	0.0038 (8)	−0.0005 (8)
C11	0.0269 (10)	0.0359 (10)	0.0287 (9)	−0.0090 (8)	0.0034 (8)	0.0026 (8)
C12	0.0172 (8)	0.0158 (7)	0.0253 (8)	0.0024 (6)	−0.0003 (7)	−0.0020 (7)

### Geometric parameters (Å, °)

**Table:**

Cl1—C3	1.7425 (18)	C5—H5	0.9300
S1—C12	1.7422 (18)	C6—C7	1.515 (2)
S1—C11	1.8092 (19)	C7—C8	1.525 (2)
S2—C12	1.6875 (17)	C7—H7	0.9800
N1—C12	1.330 (2)	C8—H8A	0.9600
N1—C9	1.481 (2)	C8—H8B	0.9600
N1—C7	1.485 (2)	C8—H8C	0.9600
C1—C2	1.383 (2)	C9—C10	1.512 (3)
C1—C6	1.394 (2)	C9—H9A	0.9700
C1—H1	0.9300	C9—H9B	0.9700
C2—C3	1.381 (3)	C10—C11	1.514 (3)
C2—H2	0.9300	C10—H10A	0.9700
C3—C4	1.379 (3)	C10—H10B	0.9700
C4—C5	1.389 (2)	C11—H11A	0.9700
C4—H4	0.9300	C11—H11B	0.9700
C5—C6	1.391 (2)		
			
C12—S1—C11	105.85 (8)	C7—C8—H8A	109.5
C12—N1—C9	124.50 (14)	C7—C8—H8B	109.5
C12—N1—C7	120.47 (14)	H8A—C8—H8B	109.5
C9—N1—C7	114.98 (13)	C7—C8—H8C	109.5
C2—C1—C6	121.49 (16)	H8A—C8—H8C	109.5
C2—C1—H1	119.3	H8B—C8—H8C	109.5
C6—C1—H1	119.3	N1—C9—C10	113.05 (14)
C3—C2—C1	119.00 (16)	N1—C9—H9A	109.0
C3—C2—H2	120.5	C10—C9—H9A	109.0
C1—C2—H2	120.5	N1—C9—H9B	109.0
C4—C3—C2	121.23 (16)	C10—C9—H9B	109.0
C4—C3—Cl1	119.37 (15)	H9A—C9—H9B	107.8
C2—C3—Cl1	119.36 (14)	C9—C10—C11	110.98 (15)
C3—C4—C5	119.06 (17)	C9—C10—H10A	109.4
C3—C4—H4	120.5	C11—C10—H10A	109.4
C5—C4—H4	120.5	C9—C10—H10B	109.4
C4—C5—C6	121.22 (16)	C11—C10—H10B	109.4
C4—C5—H5	119.4	H10A—C10—H10B	108.0
C6—C5—H5	119.4	C10—C11—S1	110.72 (12)
C5—C6—C1	117.99 (16)	C10—C11—H11A	109.5
C5—C6—C7	122.29 (15)	S1—C11—H11A	109.5
C1—C6—C7	119.64 (15)	C10—C11—H11B	109.5
N1—C7—C6	109.69 (13)	S1—C11—H11B	109.5
N1—C7—C8	110.24 (14)	H11A—C11—H11B	108.1
C6—C7—C8	115.75 (14)	N1—C12—S2	125.51 (13)
N1—C7—H7	106.9	N1—C12—S1	122.66 (13)
C6—C7—H7	106.9	S2—C12—S1	111.83 (9)
C8—C7—H7	106.9		

### Hydrogen-bond geometry (Å, °)

**Table:**

*D*—H···*A*	*D*—H	H···*A*	*D*···*A*	*D*—H···*A*
C9—H9A···S2^i^	0.97	2.85	3.773 (2)	158
C10—H10B···S2^ii^	0.97	2.77	3.701 (2)	160

Symmetry codes: (i) *x*+1/2, *y*, −*z*+1/2; (ii) −*x*, *y*+1/2, −*z*+1/2.
